# Free episomal and integrated HBV DNA in HBsAg-negative patients with intrahepatic cholangiocarcinoma

**DOI:** 10.18632/oncotarget.27002

**Published:** 2019-06-11

**Authors:** Teresa Pollicino, Cristina Musolino, Carlo Saitta, Gianluca Tripodi, Marika Lanza, Giuseppina Raffa, Francesca Casuscelli di Tocco, Chiara Raggi, Maria Consiglia Bragazzi, Adalberto Barbera, Giuseppe Navarra, Pietro Invernizzi, Domenico Alvaro, Giovanni Raimondo

**Affiliations:** ^1^ Division of Clinical and Molecular Hepatology, University Hospital of Messina, Italy; ^2^ Department of Human Pathology, University of Messina, Italy; ^3^ Department of Clinical and Experimental Medicine, University of Messina, Italy; ^4^ Humanitas Research and Clinical Center, Rozzano, Milan, Italy; ^5^ Department of Translational and Precision Medicine, Sapienza University, Rome, Italy; ^6^ Division of Surgical Oncology, University Hospital of Messina, Italy; ^7^ Present address: Department of Experimental and Clinical Medicine, University of Florence, Italy; ^8^ Present address: Division of Gastroenterology and Center for Autoimmune Liver Diseases, Department of Medicine and Surgery, University of Milano – Bicocca, Milan, Italy

**Keywords:** hepatitis B virus, covalently closed circular HBV DNA, HBV DNA integration, intrahepatic cholangiocarcinoma, occult HBV Infection

## Abstract

There is evidence that chronic hepatitis B virus (HBV) infection is associated with an increased risk of intrahepatic cholangiocarcinoma (ICC) development, and it has been hypothesized an etiological role of HBV in the development of this tumor. Very little is known about occult HBV infection (OBI) in ICC. Aims of the study were to investigate the OBI prevalence and to characterize the HBV molecular status at intrahepatic level in OBI-positive cases with ICC.

Frozen liver tumor specimens from 47 HBV surface-antigen-negative patients with ICC and 41 paired non-tumor liver tissues were tested for OBI by 4 different HBV-specific nested PCR. Covalently closed circular HBV DNA (HBV cccDNA) and viral integrations were investigated in OBI-positive cases.

HBV DNA was detected in tumor and/or non-tumor specimens from 29/47 (61.7%) ICC patients. HBV cccDNA was found in tissues from 5/17 (34.5%) cases examined. HBV integration was detected in 4/10 (40%) tumor tissues tested and involved HBx and HBV-core gene sequences in 3 and 1 cases, respectively. Viral integration occurred: (a) 9,367 nucleotides upstream of the cat-eye-syndrome critical region protein-5-isoform coding sequence; (b) within the cystinosin isoform-1-precursor gene; (c) within the thromboxane-A-synthase-1 gene; (d) within the ATPase phospholipid transporting 9B gene.

Occult HBV infection is highly prevalent in patients with ICC. Both free viral genomes and integrated HBV DNA can be present in these cases. These results suggest an involvement of HBV in the carcinogenic process leading to ICC development even in cases with occult infection.

## INTRODUCTION

Intrahepatic cholangiocarcinoma (ICC) is the second most frequent primary liver cancer after hepatocellular carcinoma (HCC) [1]. The cell of origin of cholangiocarcinoma is still a matter of debate; adult hepatocytes or cholangiocytes or stem/progenitor cells located in the canals of Hering or peribiliary glands have been considered as potential candidates. Recent reports demonstrated how ICC is specifically enriched of cells expressing markers of stem cells and co-expressing markers of the hepatocytic and cholangiocytic lineages [2–4]. In addition, common genomic traits between ICC and HCC have been identified [5–7], and gene signatures of poorly prognostic ICC are similar to those observed in poor-prognosis HCC with stem-like molecular signatures, suggesting that these distinct subgroups of liver tumors may share a common stem cell origin [8, 9]. It is well established that hepatitis B virus (HBV) is the main etiologic factor of HCC development [10]. In analogy, accumulating evidence indicates that chronic HBV infection is also associated with an increased risk of ICC development and suggests an etiological role of HBV even in the development of this tumor [11–13]. In this context, it is worth mentioning that the International Agency for Research on Cancer has recently identified ICC as an additional tumor positively linked to HBV.

HBV maintains its pro-oncogenic properties also when it is present in the liver of HBV surface antigen (HBsAg) negative patients in the so-called occult HBV infection (OBI) phase that is characterized by the long-lasting persistence at intrahepatic level of viral genomes that, despite a potentially conserved replication competence, are strongly suppressed in their activities [14]. Indeed, a large body of evidence indicates that OBI is highly prevalent in cases with HCC [14–16], whereas only a few and anecdotic reports exist on a possible involvement of OBI also in cases with ICC [17]. The aims of this study were to investigate the prevalence of OBI and to characterize the molecular status of the occult viruses in tumor and non-tumor liver specimens from ICC patients.

## RESULTS

We investigated the presence of HBV DNA sequences in liver tissue specimens from 47 HBsAg-negative patients with ICC by nested PCR and the use of 4 primer sets, each specific for preS/S, pre-C-Core, Pol, and X viral genomic regions, respectively. As we previously reported and as also recommended by others, we considered cases in which sequences of at least 2 different HBV genomic regions were detected as occult HBV-positive cases. In this way, 29 of the 47 patients with ICC (61.7%) were positive for occult HBV, and paired tumor and non-tumor tissues were available from 27 of the 29 patients.

HBV DNA sequences were revealed in the tumor tissue of 20 cases, 10 of which showed the presence of viral sequences also in the corresponding non-tumor tissue (non-tumor specimens were unavailable from 2 of the 20 cases), whereas in 9 cases viral DNA was found only in the non-tumor specimen. In particular, 6 cases showed positive reactivity for 2 or more tested HBV genomic regions both in tumor and non-tumor specimens, 9 cases only in tumor samples, and 12 cases only in non-tumor specimens (Table 1). In addition, 2 of the 6 cases from which only tumor tissue was available tested positive for occult HBV, showing positivity for 3 regions in 1 case, and for 2 regions in the remaining case (Table 1). As a note, a single HBV genomic region was detected in tumor (7 cases) or non-tumor (2 cases) tissue specimens in nine additional cases (preS/S in 3 cases; pre-C-Core in 1 case; Pol in 3 cases; X in 2 cases). The prevalence of OBI did not differ with sex or age. We found no correlation between OBI and ICC histologic characteristics (grading, necrosis, perineural and vascular invasion, lymphatic metastases; data not shown). Both the patients showing anti-HBV antibody positivity were found to be occult HBV infected, as well as 3/4 anti-HCV positive subjects.

**Table 1:** Distribution of 29 patients with ICC based on the number of positively amplified HBV genomic regions in Tumor (T) and Non-Tumor (NT) liver specimens by 4 different primer sets

No. of HBV positive regions in T/NT	4/4	3/4	2/4	4/0	0/4	1/4	2/3	0/3	2/1	1/2	2/0	0/2	3/NA	2/NA
No. of cases	3	1	1	2	1	2	1	2	1	1	6	6	1	1

NA: not available.

Paired tumor and non-tumor liver specimens for further molecular analysis were available from 17 of 27 OBI patients. By applying a highly sensitive and specific approach, HBV cccDNA was detected in tissue specimens from 5 of the 17 (34.5%) OBI patients. Among these 5 patients, cccDNA was found both in tumor and non-tumor specimens in 1 patient, and only in non-tumor tissue in 4 patients. In particular, 2 patients showed positivity for all 4 HBV genomic regions tested by PCR (1 in both tumor and non-tumor tissue and 1 only in non-tumor tissue) and 3 showed positivity for 3 HBV genomic regions. Furthermore, the Alu-PCR technique was applied to investigate the presence of HBV DNA integration in tumor tissues from 10 ICC patients. HBV integrants were detected in 4 of 10 cases examined and included a) a viral sequence containing the enhancer I/X promoter in two cases, b) a 5′-truncated HBx gene sequence including the enhancerII/basal core promoter in one case and c) a fragment of the core gene sequence in the remaining case.

**Figure 1:**
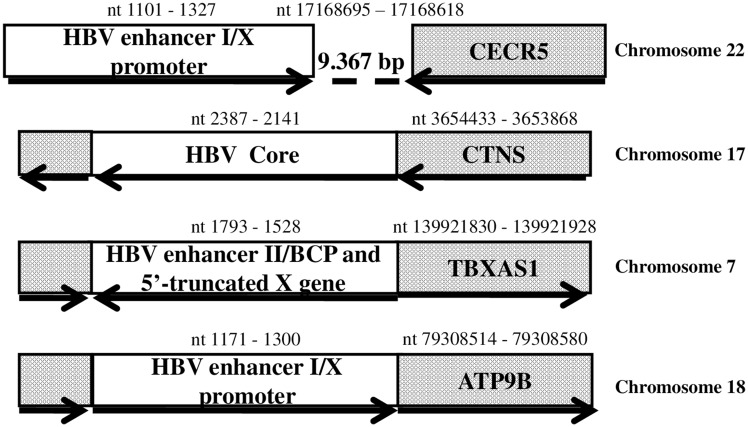
HBV DNA integrations detected in tumour samples from patients with intrahepatic cholangiocarcinoma. Dotted box: genomic coding sequence. Open box: HBV sequence. Bold arrow: open reading frame orientation. The nucleotide (nt) positions at the beginnings and ends of HBV and host genome sequences are indicated. Abbreviations: BCP, basal core promoter; bp, base pair; CECR5, cat eye syndrome critical region protein 5 isoform; CTNS, cystinosin isoform 1 precursor; TBXAS1, thromboxane A synthase 1; ATP9B, ATPase Phospholipid Transporting 9B.

The analysis of the integration sites revealed that a) the HBV sequence including the HBV enhancer I/X promoter was located 9,367 nucleotides upstream of the host genomic sequence encoding the cat eye syndrome critical region protein 5 isoform (*CECR5*); b) the core gene fragment (246 nucleotides long) was located within the cystinosin isoform 1 (*CTNS*) precursor coding sequence; c) the 5′-deleted X gene including the enhancerII/basal core promoter region was located within the coding sequence of the thromboxane A synthase 1 (*TBXAS1*); d) virus sequence including the HBV enhancer I/X promoter was located within the coding sequence of the ATPase Phospholipid Transporting 9B (*ATP9B*) (Figure 1).

The GenBank accession numbers of HBV-host integration sequences are the following: MK905389, MK905390, MK905391, MK905392.

## DISCUSSION

Although in more than 50% of cases ICC arises de novo without any identifiable risk factor and in the context of apparently normal livers, a significant association of ICC development with chronic HBV and HCV infections has recently emerged [11–13, 18, 19]. In accordance with the epidemiology of these viruses, the association with HCV is evident in Western countries and in Japan [20–22], whereas HBV is clearly a major risk factor for ICC occurrence in China, South Korea and Taiwan [11, 23, 24]. This scenario exactly reflects the epidemiology of hepatitis viruses as well as the geographic distribution of HBV- and HCV-related HCC.

We found a very high prevalence of OBI in ICC patients from different Italian regions. According to the same criteria of at least 2–4 positive HBV genomic regions tested, by PCR this prevalence is comparable with that reported in several studies investigating OBI in HCC cases [25–27]. In particular, such a prevalence is very similar to that found in a previous study that examined a large cohort of HBsAg-negative patients from different Italian regions with both HCV-related and cryptogenic HCC [14]. Furthermore, in the present study we observed that HBV can be present in ICC as episomal cccDNA as well as integrated into the host’s genome. Altogether, these data indicate that HBV is highly prevalent in patients with primary liver cancers (ICC as well as HCC). Considering that the HBsAg prevalence in the Italian general population is estimated to be lower than 1% since at least the beginning of this century [28], our results might lead to speculate that HBV is a major hepatocarcinogenic player even in areas where it is not endemic. Indeed, our results are quite unexpected and their novelty leads to some considerations and to imagine new possible routes for future research in this field. Na^+^-dependent taurocholate transporter (NTCP, gene *SLC10A1*) is currently considered the transporter involved in HBV entrance in hepatocytes. As a consequence, only cells expressing NTCP could be infected. Adult mature cholangiocytes do not express NTCP and therefore this is against a possible origin of HBV positive (including OBI positive) ICC from adult cholangiocytes. Though, it cannot be ruled out that other transporters expressed in cholangiocytes might be involved in HBV entry under peculiar circumstances. During the differentiation of resident stem/progenitor cells toward adult hepatic cells, NTCP starts to be expressed in the stage of hepatoblasts, cells with a bipotential differentiative capacity toward the hepatocytic or cholangiocytic lineage [29, 30]. The observation that a significant proportion of ICC cells co-express markers of the hepatocytic or cholangiocytic lineage [2–4] suggests that these cells (bipotential progenitors) could be infected by HBV, thus representing the cell of origin of ICC. Therefore, it could be hypothesized that the neoplastic transformation of HBV-infected bipotential progenitors, typically blocking the processes of cell differentiation, promotes the development of a neoplasm enriched in cancer stem cells (i.e. cholangiocarcinoma [4–6]) rather than HCC that, in contrast, originates almost exclusively in cirrhotic livers from adult infected hepatocytes. Indeed, more than 90% of the analyzed ICC samples were from patients without liver cirrhosis. As an alternative, the trans-differentiation of adult HBV-infected hepatocytes into cells of the biliary lineage could be taken into consideration for the origin of ICC-OBI.

Indeed, the presence of HBV DNA in a considerable number of ICC specimens might suggest that these tumors derive from the transformation of hepatocyte precursors prone to infection with HBV, and potentially support its replication cycle. It has been suggested that hepatocyte precursor cells may transform into HCC or trans-differentiate into cells of the biliary lineage, which can acquire a malignant phenotype generating ICC.

Future research would clarify whether these different liver cancers share a common molecular origin and whether (and how) the oncogenic properties of HBV are involved in their development. In addition, the finding of viral DNA integration into genes known to be involved in cancerogenesis [31–34] strongly suggest extending the studies on HBV integration also to patients with ICC, both with overt and occult infection, by the use of the most sensitive and specific technical approaches such as the next generation sequencing based methods. These studies might provide fundamental information on the pathogenesis of ICC and the possible role exerted by HBV integrants in promoting this cancer.

## MATERIALS AND METHODS

### Patients

Frozen liver tumor specimens from 47 HBsAg-negative patients with ICC (23 men and 24 women; mean age 63.8 ± 10.9 years) and 41 paired non-tumor liver tissues were studied. The patients had consecutively undergone surgical resection between 2009 and 2015 in three Italian liver centres located in distinct geographic areas of the country (Milan, Rome, and Messina). Liver specimens had been frozen and stored in liquid nitrogen immediately after surgery and never thawed before this study. Furthermore, expert pathologists performed histological diagnosis of ICC and co-existence of HCC features or combined hepatocellular- cholangiocarcinoma have been excluded in all the cases. Liver histology examination of non-tumor tissues showed minimal changes (F0-F1 at Metavir score) in 23/41 cases, chronic hepatitis (F2-F3) in 14/41, and cirrhosis (F4) in 4/41 cases. Four of the 47 patients (8.5%) were antibody to hepatitis C virus (anti-HCV) positive (Table 2). Data on circulating anti-HBV antibodies were available in 12 cases. Two of them (16.7%) were antibody to HBV core antigen (anti-HBc) positive (both of whom were also positive for antibody to HBsAg [anti-HBs]), while the remaining 10 cases were negative for all HBV serum markers. None of the patients had primary sclerosing cholangitis. Four of the 41 (9.8%) patients from whom non-tumor liver tissues were available for histological evaluation had non-alcoholic steatohepatitis. The study protocol was approved by local ethical committees, performed according to the principles of the Declaration of Helsinki, and informed consent was obtained from all patients.

**Table 2:** Demographic and histological characteristics of 47 HBsAg-negative patients with ICC

**Male sex, *n* (%)**	23 (48.9)
**Age (mean ± SD, years)**	63.8 ± 10.9
**Anti-HCV positive patients, *n* (%)**	4 (8.5)
**Non-tumor liver histology**^†^ **(Metavir score)**	
F0-F1 *n*. (%)	23 (56.1)
F2-F3 *n*. (%)	14 (34.1)
F4 *n*. (%)	4 (9.8)

^†^Non-tumor tissues available from 41 patients.

### HBV DNA analyses

All frozen tumor specimens were tested for occult HBV DNA through previously described methods based on nested PCR amplification [14, 35]. Briefly, DNA was extracted from each specimen by means of the Master-Pure TM DNA Purification Kit (epicentre, Madison, WI, USA). All liver DNA extracts were analysed for the presence of HBV genomes by performing four different in-house nested PCR amplification assays to detect preS-S, pre-C-Core, Pol, and X viral regions, respectively. Appropriate negative and positive controls were included in each PCR experiment. In particular, the following negative controls were included in each test (i) liver DNA extracts from specimens known to be HBV DNA negative; (ii) DNA-free reaction buffer; (iii) water. In addition, to eliminate false negative results, beta-globin was used as a housekeeping gene. The lower detection limit of our nested PCR amplification was 10 genome equivalents/mL for all primer sets used. Moreover, Southern blot analysis and direct sequencing of all amplified HBV sequences confirmed the specificity of the reactions. The cases that showed positivity in at least 2 different viral genomic regions were considered HBV DNA positive [35].

### Detection of HBV Covalently Closed Circular DNA

HBV cccDNA was tested in OBI positive patients from whom paired tumor and non tumor liver specimens were available. The Hirt procedure was used to isolate HBV cccDNA [36]. Briefly, liver biopsy samples were homogenized with a Tissue ruptor device in 500 μl homogenization buffer, lysed in the presence of 2% SDS, precipitated by an overnight incubation in high salt buffer (final concentration 0.5 M KCl), and centrifuged. Viral DNA in the supernatant was extracted with phenol/chloroform followed by an ethanol precipitation. The Hirt-extracted DNA was treated with T5 exonuclease (New England Biolabs) to digest linear and relaxed circular DNA (rcDNA) according to the manufacturer’s instructions. Nested PCR for HBV cccDNA detection was performed as previously described [14]. In particular, the primers used [sense primers HBV P23 and HBV P25, located within the single-stranded region of rcHBV, used together with the antisense primers HBV P24 and HBV P26 (Table 3)] were selected to discriminate between rcDNA and cccDNA present in the infected cells. The limit of sensitivity of nested PCR for cccDNA detection was 10 genome equivalents/mL.

**Table 3:** Oligonucleotides used as PCR primers to detect HBV cccDNA

	Name	Nucleotide sequence	Position†
Sense primers	HBV P23	5′-CTGAATCCTGCGGACGACCC-3′	1441–1460
	HBV P25^‡^	5′-GTCTGTGCCTTCTCATCTGCC-3′	1551–1571
Antisense primers	HBV P24	5′-CCCAAGGCACAGCTTGGAGG-3′	1889–1869
	HBV P26^‡^	5′-AGAGATGATTAGGCAGAGGTG-3′	1846–1826

^†^Nucleotide positions of the primers are numbered from the unique HBV EcoRI site, and the nomenclature is according to Galibert *et al*. [38]

^‡^Applied in the second round of amplification of the nested PCR.

### Detection of HBV DNA integration into the host genome

We assayed the presence of integrants in the liver DNA extracts testing positive for HBV sequences, including those reacting positive for only one of the four different HBV genomic regions analyzed. HBV DNA insertion into the hepatocyte genome was investigated by applying the Alu-PCR technique in accordance with described methods [37]. Briefly, amplification was carried out in a final volume of 50 μl, containing 100 ng of genomic DNA as a template, 10 pM of Alu primer and 100 pM of HBV primers designed for three distinct viral genomic regions (Core, X and preS/S). A hot start technique was used and one unit of uracil DNA glycosylase was added to each tube after the first 10 cycles of amplification; the tubes were incubated for 30 minutes at 37° C and heated for 10 minutes at 94° C to break the DNA strands at apurinic dUTP sites. A ‘touchdown’ PCR technique was then employed for a total of 40 cycles. Five microliters of the amplified products were subjected to nested PCR with internal primers. In order to confirm the specificity and increase the sensitivity of the PCR results, Southern Blot analysis was performed on all the amplified products following standardized procedures. Nucleotide sequences of the PCR products containing viral-host junctions were determined by direct sequencing and the use of the BigDye Terminator Cycle Sequencing Ready Reaction Kit (Applera, Foster City, CA, USA) according to the manufacturer’s instructions. The sequencing products were resolved in an automatic DNA sequencer (ABI PRISM 3500 Dx Genetic Analyzer; Applera). Samples that tested negative after PCR amplification on agarose gel, but subsequently tested positive for HBV DNA integration by Southern Blot analysis were cloned into the PCR-TOPO vector (Invitrogen, Milan, Italy). Single clones were selected for sequencing, as described above. Nucleotide sequences were assessed using the BLAST search system. HBV integrated sequences were then better characterized using the CLUSTAL W program.

## Abbreviations

ICC: intrahepatic cholangiocarcinoma
HCC: hepatocellular carcinoma
HBV: hepatitis B virus
HBsAg: HBV surface antigen
OBI: occult HBV infection
anti-HCV: antibody to hepatitis C virus
anti-HBc: antibody to HBV core antigen
anti-HBs: antibody to HBsAg
cccDNA: covalently closed circular DNA
rcDNA: relaxed circular DNA
*CECR5*: cat eye syndrome critical region protein 5 isoform
*CTNS*: cystinosin isoform 1
*TBXAS1*: thromboxane A synthase 1
*ATP9B*: ATPase Phospholipid Transporting 9B
NTCP: Na^+^-dependent taurocholate transporter
